# A population-based observational study comparing Cervista and Hybrid Capture 2 methods: improved relative specificity of the Cervista assay by increasing its cut-off

**DOI:** 10.1186/s12879-014-0674-1

**Published:** 2014-12-09

**Authors:** Gerd Boehmer, Lisa Wang, Angelika Iftner, Barbara Holz, Juliane Haedicke, Reinhard von Wasielewski, Peter Martus, Thomas Iftner

**Affiliations:** German Clinic Bad Münder, Bad Münder, Germany; Clinical Epidemiology and Applied Biometry, University Hospital Tübingen, Tübingen, Germany; Division of Experimental Virology, Institute of Medical Virology, University Hospital Tübingen, Elfriede-Aulhorn-Str. 6, Tübingen, 72076 Germany

**Keywords:** Cervista, Hybrid capture, HPV, Cervical cancer screening

## Abstract

**Background:**

High-risk human papillomavirus (HR HPV) testing has been shown to be a valuable tool in cervical cancer screening for the detection of cervical pre-cancer and cancer.

**Methods:**

We report a purely observational study evaluating HR HPV prevalences in residual liquid-based cytology (LBC) samples using both the Cervista™ HPV HR Test and the Digene Hybrid Capture 2 High-Risk HPV DNA Test (HC2) in a sample of 1,741 women aged ≥30 years of a German routine screening population of 13,372 women. Test characteristics were calculated and a novel method for measuring test performances was applied by calculating ratios of sensitivity or specificity.

**Results:**

The overall agreement of both tests for detection of HR HPV was excellent (κ = 0.8). Relative sensitivities for the detection of histologically confirmed severe cervical intraepithelial dysplasia (CIN3+) were similar for both HPV-tests, which was confirmed by the ratio analysis. However, discrepancy analysis between the Cervista HPV HR test and HC2 revealed a high false positive rate of the Cervista HPV HR test in the cytology normal category.

**Conclusions:**

Performance of the Cervista HPV test in cervical specimens with abnormal cytology is comparable to HC2 as both tests were highly sensitive and specific for the detection of high grade cervical disease. We also demonstrate evidence that modification of the cut-off values drastically reduces the false positive rate in the cytology normal category without affecting the detection of CIN3+, which ultimately improved specificity of the Cervista HPV HR assay.

**Electronic supplementary material:**

The online version of this article (doi:10.1186/s12879-014-0674-1) contains supplementary material, which is available to authorized users.

## Background

In Germany, the cervical cancer mortality rate has notably decreased since the introduction of gynaecological screening for cervical cancer in 1971 [1]. Annual opportunistic screening is usually performed by conventional cytology (Pap-smear) and is covered by health insurances for women aged 20 years or older. Nevertheless, 4,900 new cases and approximately 1,600 deaths of cervical cancer are observed each year [2] and 150,000 cases of cervical cancer precursors (CIN3) are diagnosed [3]. Cervical cancer accounts for 1.6% of all cancer deaths among women in Germany [2].

Persistent infection with high-risk human papillomaviruses (HR HPV) has been shown to be necessary for the development of cervical precancerous lesions and cancer [4]. Notably, the HR HPV types 16, 18, 31, 33, 35, 39, 45, 51, 52, 56, 58 and 59 have been defined as class I carcinogens and HPV type 68 as class IIa carcinogens by the IARC [5]. The majority of all cervical cancer cases are associated with the HR HPV types 16 and 18 [6]. The fact that HR HPV is the causative infectious agent of cervical cancer has led to the development and investigation of various HPV detection methods; and testing for HR HPV in addition to cytology is nowadays applied in cervical cancer screening [7]-[9]. Three DNA-based tests for the detection of the HR HPV group and one RNA-based assays for HPV detection have been approved by the US Food and Drug Administration (FDA) for routine cervical cancer screening (http://www.fda.gov/MedicalDevices/ProductsandMedicalProcedures/InVitroDiagnostics/ucm330711.htm). Among these are the Digene Hybrid Capture 2 High-Risk HPV DNA test (HC2; QIAGEN, Hilden, Germany), the Cervista HPV HR test (CER; Hologic, Madison, WI), the cobas*®* HPV Test (Roche*,* Pleasanton, USA) and the RNA-based APTIMA® HPV Assay (Hologic, San Diego, CA). The cobas® HPV Test has recently been approved for primary screening by the FDA (www.fda.gov).

The HC2 test for the collective detection of at least 13 carcinogenic HPV types (16, 18, 31, 33, 35, 39, 45, 51, 52, 56, 58, 59, 68) [10] is a nucleic acid hybridisation assay with signal amplification using microplate chemiluminescence for semi-quantitative detection of HPV-DNA in cervical specimens. In addition to the 13 carcinogenic HPV types detected by HC2, the Cervista HPV HR assay also detects putative HR HPV type 66 [11]. The test principle employs the Invader® chemistry, which is a signal amplification method recognizing specific nucleic acid sequences.

Performance comparisons of HR HC2 and the Cervista HPV HR test have previously been performed [12]-[22]. However, only a limited number of studies present data from both assays on the same residual LBC sample in comparison to cytology and histology results [12],[16],[17],[19]. The objective of this study was therefore to evaluate the Cervista HPV HR assay in comparison to HC2 regarding relative sensitivity and specificity for the detection of high grade CIN3+ in women of a German routine screening population aged ≥30 years using cervical samples collected in PreservCyt® LBC medium. PreservCyt® LBC cervical samples from 1,741 women with abnormal (n = 468), ASC-US (n = 20) as well as a random sample of women with normal cytology (n = 1,208) were analyzed by the Cervista HPV HR test and HC2.

## Methods

### Study design

**Figure 1:**
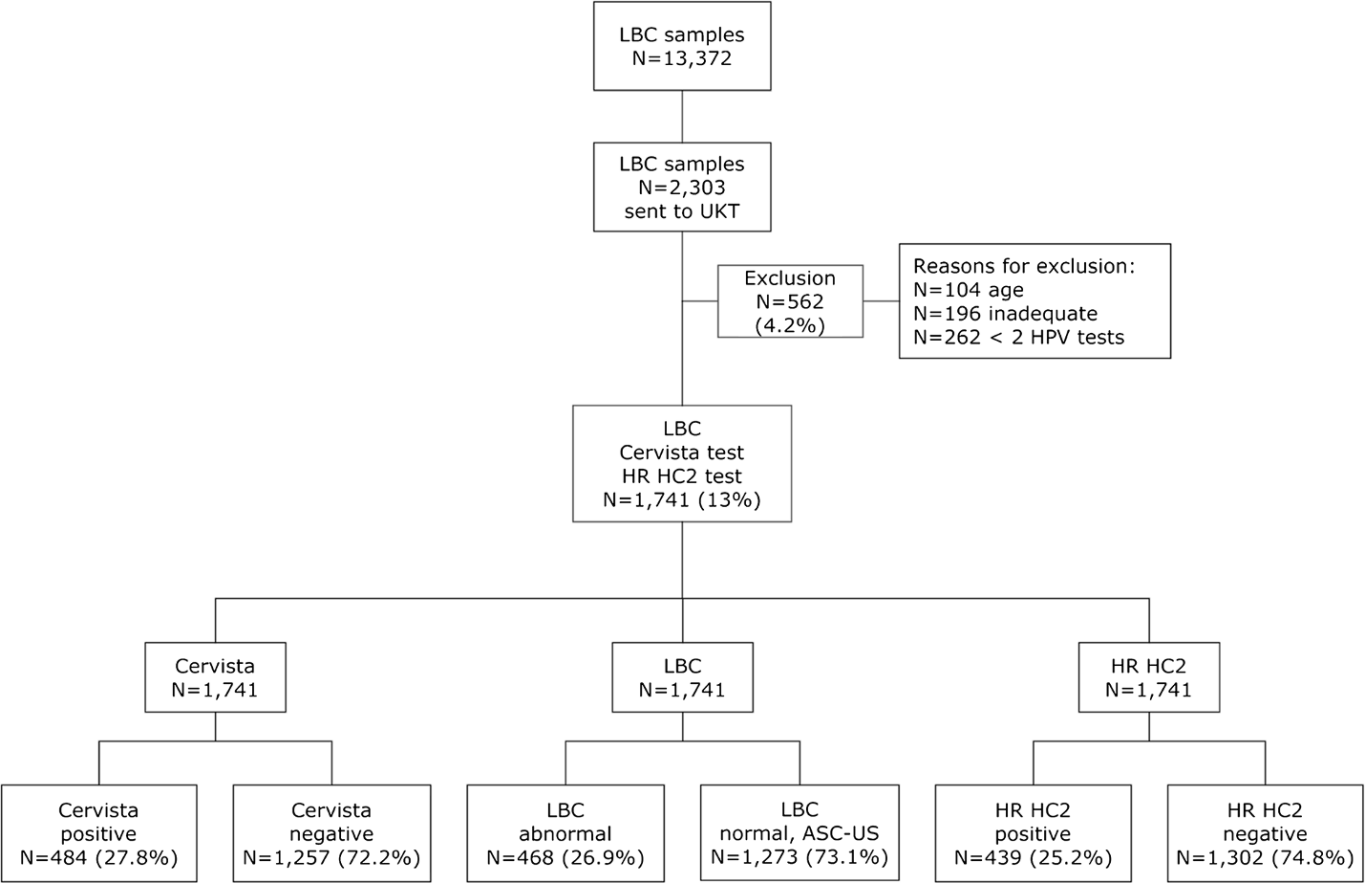
**Study design flowchart.**

The study was conducted in a routine screening population of 13,372 women ≥ 30 years of age living in the Hannover area of Germany (Figure 1). Cervical samples were collected in PreservCyt® Pap Test specimen collection medium (Hologic, Marlborough, MA) between February and June 2011 and cytology was tested within one week after collection in a central services laboratory (Amedes, Bad Münder, Germany). As determined by the ethics committee (Ethikkommission bei der Deutschen Ärztekammer Niedersachsen), no ethical approval was required for this purely observational study, because residual patient samples were used that were completely anonymized and study results had no influence on the patients’ follow-up strategy. A total of 2,303 liquid based cytology (LBC) smears including all residual samples with abnormal and ASC-US cytology results as well as 10% of randomly selected normal LBC samples were sent to Tübingen for HPV testing.

After exclusion of ineligible samples a total of 1,741 specimens from women with abnormal, ASC-US and normal cytology were analyzed both by the Cervista™ HPV HR assay and by the Digene Hybrid Capture 2 High Risk DNA Test. This collection also included 60 samples of patients with CIN3+, which were added to the cohort in order to obtain a higher CIN3+ rate. Samples with discordant HPV test results were genotyped by INNO-LiPA HPV Genotyping Extra® for discrepancy analysis. Participants were blinded for their HPV test results, and colposcopy and histopathology was only performed when indicated. Samples with a primary histology of CIN2+ were independently reviewed.

### Sample collection

According to routine guidelines all clinical samples were collected in LBC PreservCyt® Collection medium (Hologic) using the Cervex broom and were subsequently tested by cytology in the central cytology laboratory within one week of collection.

### Liquid based cytology

All samples were tested by the ThinPrep® 2000 Processor (Hologic) following the manufacturer’s instructions. Cytology results were reported using the Munich Nomenclature II and were translated into The Bethesda System (TBS) (Table 1). LBC results were considered negative when the result was Pap I/II or Pap IIw; all other results were considered positive.

**Table 1:** **Results LBC versus histology and HPV testing**

		Primary Histology (N = 139)					
	Not performed	Normal	Borderline	CIN1	CIN2	CIN3+	Total
**LBC**	N(Cer+,HC2+)	N(Cer+,HC2+)	N(Cer+,HC2+)	N(Cer+,HC2+)	N(Cer+,HC2+)	N(Cer+,HC2+)	N(Cer+, HC2+)
**Normal** (Pap I/II)	1,208 (130, 69)	0	0	0	0	0	1,208 (130, 69)
**ASC-US** (Pap IIw)	65 (20, 18)	0	0	0	0	0	65 (20, 18)
**ASC-H, AGUS** (Pap III)	14 (6, 5)	0	1 (0, 0)	0	0	5 (4, 4)	20 (10, 9)
**LSIL, HSIL** (Pap IIID)	312 (197, 211)	21 (16, 18)	1 (1, 1)	5 (4, 4)	13 (12, 13)	20 (20, 20)	372 (250, 267)
**HSIL, CIS** (Pap IVa)	3 (3, 3)	1 (1, 1)	0	3 (3, 3)	6 (6, 6)	58 (56, 58)	71 (69, 71)
**HSIL, CIS, Micro** (IVb)	0	0	0	0	0	2 (2, 2)	2 (2,2)
**Microinvasive** (Pap V)	0	0	0	0	0	3 (3, 3)	3 (3, 3)
**Total**	1,602 (356, 306)	22 (17, 19)	2 (1, 1)	8 (7, 7)	19 (18, 19)	88 (85, 87)	1,741 (484, 439)

Cer+: Cervista positive; HC2+: HC2 positive.

### DNA extraction and HPV testing

Residual LBC samples were sent to the central molecular testing laboratory (UKT, Tübingen) within 2 weeks of collection for HPV testing. For testing by Cervista™ HPV HR assay, DNA extraction was performed on 2ml of each ThinPrep sample using the GenFind® DNA Extraction Kit in combination with the ThinPrep 5000 STS (Sample Transfer System, (Hologic). To test for a possible risk of cross-contamination during sample transfer by the STS, we ran a pilot experiment with alternating blank samples and samples containing HPV16-positive SiHa cells and found no evidence of cross-contamination. DNA extraction was carried out according to the manufacturer’s instructions. DNA integrity was measured by PCR using primers targeting the human β-globin gene. Using the Cervista high throughput automation (HTA) system DNA samples (10 μl per reaction) were then tested by Cervista™ HPV HR assay, which was performed in compliance with the manufacturer’s instructions. The current cut-off definitions were used in this study, which were calculated as per the manufacturer’s instructions. Briefly, a signal-to-noise value was generated for each of three reactions, which is referred to as Fold-Over-Zero (FOZ). A FOZ ratio was calculated by dividing the highest by the lowest FOZ value of the three reactions. A given sample was considered HPV-positive if the HPV FOZ ratio was ≥1.525. When the FOZ ratio was <1.525, but all three individual reaction FOZ values were ≥1.93, the sample was also considered positive for HPV.

4ml of each ThinPrep sample were processed for HR HC2 testing, which was performed using the Rapid Capture System 1 (RCS-1) according to the manufacturer’s instructions. The cut-off value of RLU/CO = 1, equivalent to 1pg HPV DNA per 1ml of sampling buffer for positive test results, was used in this study. PreservCyt® specimens were retested when RLU/CO ratios between ≥1.0 and <2.5 were obtained. If the initial retest result was positive (RLU/CO of ≥1.0), the specimen was reported as “positive”. If the retest was negative (RLU/CO of <1.0), a second repeat test (third result) was performed to generate a final result.

HPV genotyping was carried out using the INNO-LiPA HPV Genotyping Extra® test as previously described [23].

### Histology review

All samples with a primary histology result of CIN2+ were reviewed by an independent external expert. In case of a discrepant review reading, a second histology review was performed. If two out of three diagnoses were identical, the result was considered final.

### Statistical analysis

Statistical analysis was performed on all samples with valid test results from LBC, the Cervista HPV HR test and HC2 (N = 1,741). Cohen’s kappa value (κ) was used to calculate the agreement between the Cervista HPV HR test and the HC2 test. 95% confidence intervals (CI) for HPV prevalence were calculated using the Wilson score method. McNemar’s test for comparison of two proportions was used for calculating two-sided P-values to assess statistical significance of different Cervista HPV HR and HC2 test results. In addition, relative sensitivities and specificities as well as positive predictive values (PPV) and negative predictive values (NPV) based on cytological results and histology were calculated according to Cuzick et al. [24]. The statistics software package R version 3.0.2. was used for statistical analyses.

### Ratio analysis of sensitivities and specificities

Relative performance of the tests were measured by calculating the ratio of sensitivity and the ratio of specificity. The ratio of two sensitivities is the True Positive rate of test 1 divided by the True Positive rate of test 2. The ratio of two specificities depends on the prevalence, which in this case was Spec(CER)/Spec(HC2) = (0.77 – Prevalence)/(0.80 – Prevalence). A full description of this method can be found in the Additional file 1. Confidence intervals were calculated using the delta method transform on the proportion’s confidence interval. The ratio analysis has been performed using the statistics software package R version 3.0.2.

## Results

Out of 2,303 overall specimens, a total of 562 were excluded due to inadequacy (n = 196), insufficient material (n = 262) or because study participants were younger than 30 years (n = 104; Figure 1). The 1,741 remaining specimens were analysed by both the Cervista HPV HR assay and HC2. 468 of these samples had an abnormal cytology result (Pap ≥ III), 65 were classified ASC-US (Pap IIw) and 1,208 women were cytologically normal (Pap I/II) (Table 1). Colposcopy and histopathology was performed on 139 patients with abnormal cytology results and was not performed on cytologically normal women due to the purely observational study design. A high CIN3+ rate was obtained by including 60 additional patients with confirmed CIN3+.

### HPV prevalence detected by the Cervista HPV HR test and HC2

Concordant results were obtained from 1,608 (92.4%) of 1,741 LBC samples (Table 2). The agreement between the tests was excellent at κ = 0.8 (95% CI: 0.77-0.84). Overall HPV detection rates by the Cervista HPV HR test and HC2 for all LBC categories are summarized in Table 3. In women with normal LBC results, HR HPV was detected in 10.8% of the specimens by the Cervista HPV HR test and in 5.7% using the HC2 test (κ = 0.53 (0.43-0.61)). HPV prevalence detected in the ASC-US category (Pap IIw) was 30.8% for the Cervista HPV HR assay and 27.7% for HC2, respectively. For category ASC-H and AGUS (Pap III), Cervista HPV HR test-positivity was 50% compared to 45% for HC2; for LSIL, HSIL (Pap IIID) 67.2% and 71.8% and for HSIL, CIS (Pap IVa) 97.2% and 100%, respectively. 100% HPV-positivity was determined for both tests in the cytology categories HSIL (Pap IIID), CIS (IVb) and microinvasive (Pap V). Interestingly, kappa values (κ) calculated to measure the agreement of both HPV tests were only fair (κ ≤ 0.55) for LBC categories ≤ ASC-US (≤Pap IIw). For categories AGUS+ (≥Pap III) the agreement was excellent (0.83 (0.77-0.88); Table 3).

**Table 2:** **Comparison of Cervista HPV HR test and HC2 results**

		HR HC2		
		Positive	Negative	Total
		(N/%)	(N/%)	(N/%)
	**Positive**	395/22.7%	89/5.1%	484/27.8%
**CER**	**Negative**	44/2.5%	1,213/69.7%	1,257/72.2%
	**Total**	439/25.2%	1,302/74.8%	1,741/100%

The overall agreement between Cervista (CER) and HC2 test results was κ = 0.8 (95% CI: 0.77-0.84).

**Table 3:** **HPV prevalence detected by Cervista and HC2 in comparison to LBC**

LBC	CER+ve (%)	HR HC2+ve (%)	κ (95% CI)
**Normal** (Pap I/II)	10.8%	5.7%	0.53 (0.43-0.61)
**ASC-US** (Pap IIw)	30.8%	27.7%	0.55 (0.33-0.78)
**ASC-H, AGUS** (Pap III)	50.0%	45.0%	0.9 (0.71-1.0)
**LSIL, HSIL** (Pap IIID)	67.2%	71.5%	0.82 (0.75-0.88)
**HSIL, CIS** (Pap IVa)	97.2%	100 %	1
**HSIL, CIS, Micro** (IVb)	100%	100%	1
**Microinvasive** (Pap V)	100%	100%	1
**AGUS+** (≥Pap III)	71.3%	75.2%	0.83 (0.77-0.88)
**HSIL+** (≥Pap IVa)	100%	100%	1
**Total**	27.8%	25.2%	0.8 (0.77-0.84)

+ve: positive; +: and higher.

95% CI for HPV prevalence were calculated using the Wilson Score method.

### Discrepancy analysis

A total of 133 discordant samples were detected between the Cervista HPV HR test and HC2 (Table 4). 89 discrepant samples (66.9%) were Cervista HPV HR test-positive and HC2-negative compared to only 44 samples (31.7%) with HC2-positive and Cervista-negative test results. The majority of discordant results (n = 89) was detected in specimens with normal cytology, followed by the LSIL and HSIL category. Within the Pap normal category, 84.3% of the discordant samples were CER- positive and HC2-negative in contrast to the PapIIID (LSIL, HSIL) group where 79.3% of the discordant samples were positive by HC2 and negative by Cervista HPV HR-test results.

**Table 4:** **Distribution of discordant results by LBC results**

LBC	HR HC2-ve, CER+ve (N/%)	CER-ve, HR HC2+ve (N/%)	Total (N)
**Normal** (Pap I/II)	75/84.3%	14/15.7%	89
**ASC-US** (Pap IIw)	7/58.3%	5/41.7%	12
**ASC-H, AGUS** (Pap III)	1/100.0%	0	1
**LSIL, HSIL** (Pap IIID)	6/20.7%	23/79.3%	29
**HSIL, CIS** (Pap IVa)	0	2/100.0%	2
**Total**	89	44	133

+ve: positive; −ve: negative.

By genotyping of all deviant samples (n = 133) using the INNO-LiPA HPV Genotyping Extra test (Table 5), we resolved 56 HC2-negative and 3 CER-negative samples as true negatives revealing an unexpectedly high number of false positive results for Cervista (n = 56). While a total of 21 samples were inadequate for LiPA genotyping, all remaining samples (n = 53) were HPV DNA positive. In detail, 16 specimens missed by Cervista contained non-target types of the Cervista HPV HR assay (including 2 HPVX types) and a total of 23 samples (52.3%) were false-negative by Cervista. These included 3 specimens with CIN2+ histology. In contrast, 7 discordant samples (7.9%) with negative HC2 and positive Cervista HPV HR test results were false-negative by HC2, while another 7 samples contained non-target types of the HC2 test (including 2 HPVX types). However, none of the samples missed by HC2 had a histology result of CIN2+.

**Table 5:** **Discordant HPV test results resolved by their HPV genotype detected by INNO-LiPA genotyping**

HPV genotype	HPV classification	HR HC2-ve, CER+ve (N)	CER-ve, HR HC2+ve (N)	Histology CIN2+ (HC2-ve/CER-ve)
HPV16*	HR	4	4	0/2
HPV18*	HR	0	1	0/1
HPV31*	HR	0	3	0
HPV33*	HR	0	1	0
HPV39*	HR	1	1	0
HPV51*	HR	1	6	0
HPV52*	HR	1	4	0
HPV66**	HR	2	1	0
HPV68*	Intermediate	0	2	0
**HPV53**	Intermediate	1	11	0
**HPV6**	LR	0	1	0
**HPV54**	LR	0	1	0
**HPV69/71**	LR	1	0	0
**HPV74**	LR	1	1	0
**HPVX**		2	2	0
**HPV DNA negative**		56	3	0
**no result** **(sample failed)**		19	2	0
**Total**		89	44	3

*HC2 and Cervista target types; **Cervista target type; HPVX: HPV DNA was detected by LiPA, but could not be correlated to a specific type; +ve: positive; −ve: negative.

### Sensitivity and specificity

Relative sensitivity and specificity were calculated in order to measure clinical outcomes related to HPV test results. A total of 139 biopsies were taken from patients with abnormal cytology when indicated and analysed by histopathology (Table 1). Overall relative sensitivity for detection of histologically confirmed CIN3+ in patients with abnormal cytology (AGUS+) was 96.6% for the Cervista HPV HR test and 98.9% for the HC2 test (Table 6). Relative specificity and PPV for Cervista HPV HR test were 15.7% and 66.4% or 9.8% and 65.4% for HC2 test, respectively.

**Table 6:** **Relative sensitivity, relative specificity, PPV and NPV of the Cervista HPV HR test and HC2 for detecting CIN3+ within various cytology groups**

	CERVISTA							
	Relative sensitivity		Relative specificity		PPV		NPV	
CIN 3+	%	95% CI	%	95% CI	%	95% CI	%	95% CI
**ASC-H, AGUS** (Pap III)	80.0%	(29.9-98.9)	100.0%	(54.6-100)	100.0%	(39.6-100)	50.0%	(26.7-97.3)
**LSIL, HSIL** (Pap IIID)	100.0%	(80.0-100)	17.5%	(7.9-33.4)	37.7%	(25.1-52.1)	100.0%	(56.1-100)
**HSIL, CIS** (Pap IVa)	96.6%	(87.0-99.4)	NA		84.8%	(73.4-92.1)	NA	
**HSIL, CIS, Micro** (IVb)	100.0%	(19.8-100)	NA		100%	(19.8-100)	NA	
**Microinvasive** (Pap V)	100.0%	(30.1-100)	NA		100%	(30.1-100)	NA	
**AGUS+** (≥Pap III)	96.6%	(89.7-99.1)	15.7%	(7.5-29.1)	66.4%	(57.5-74.4)	72.7%	(39.3-92.7)
**HSIL+** (≥Pap IVa)	96.8%	(88.0-99.4)	NA		85.9%	(75.2-92.7)	NA	
	**HR HC2**							
	**Relative sensitivity**		**Relative specificity**		**PPV**		**NPV**	
**CIN 3+**	**%**	**95% CI**	**%**	**95% CI**	**%**	**95% CI**	**%**	**95% CI**
**ASC-H, AGUS** (Pap III)	80.0%	(29.9-98.9)	100.0%	(54.6-100)	100.0%	(39.6-100)	50.0%	(26.7-97.3)
**LSIL, HSIL** (Pap IIID)	100.0%	(80.0-100)	10.0%	(3.3-24.6)	35.7%	(23.7-49.7)	100.0%	(39.6-100)
**HSIL, CIS** (Pap IVa)	100.0%	(92.3-100)	NA		85.3%	(74.2-92.3)	NA	
**HSIL, CIS, Micro** (IVb)	100.0%	(19.8-100)	NA		100%	(19.8-100)	NA	
**Microinvasive** (Pap V)	100.0%	(30.1-100)	NA		100%	(30.1-100)	NA	
**AGUS+** (≥Pap III)	98.9%	(92.9-99.9)	9.8%	(3.7-22.2)	65.4%	(56.6-73.3)	83.3%	(36.5-99.1)
**HSIL+** (≥Pap IVa)	100.0%	(92.8-100)	NA		86.3%	(75.8-92.9)	NA	

+: and higher.

**Figure 2:**
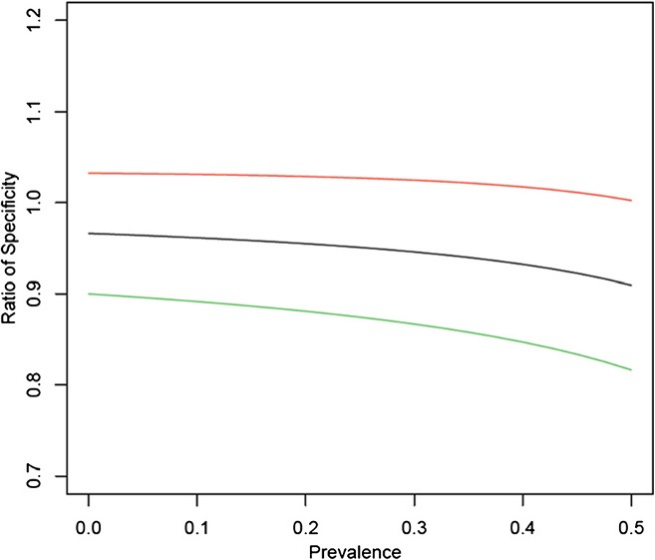
**Ratio of specificities of Cervista and HC2 as a function of HR HPV prevalence.**

As only women with high grade abnormal cytology were referred to colposcopy and considering the low sensitivity of cytology in detecting CIN3+ [25], the true extent of high grade CIN in the Pap I/II and IIw categories remains unknown. This lack of data prevents the application of conventional clinical sensitivity and specificity calculations. However, by calculating the ratio of the sensitivity or specificity of two tests, we developed a method, which yields a value that measures relative performance. The ratio of sensitivity between Cervista HPV HR test and HC2 is 0.977 (0.69, 1.27). The ratio of specificity also contained 1 over all prevalence possibilities. Figure 2 shows the ratio of specificity given a range of prevalences. As a result, there is no statistical difference between clinical sensitivity and specificity of the Cervista HPV HR test and HC2 for the detection of high grade disease at the 95% confidence level.

N = 100 histology reviews of slides with a primary histology result of CIN2+ were performed in the 19 CIN2 samples and in 81 of the 88 CIN3+ samples. Due to the review 5 samples changed from CIN2 to the final result CIN3 and one sample changed from a primary histology result CIN3 to a final histology CIN2. Sensitivity and specificity results for the performance of the Cervista HPV HR test versus HC2 with regard to the endpoint CIN3+ did not change when the review results were accounted for in the calculations (data not shown).

### Different Cervista cut-off values reduce HPV-positivity without affecting detection of CIN3+

We found a significantly higher positivity rate of the Cervista HPV HR test (10.8% positive results for Cervista versus 5.7% for HC2; p <0.0001) in cytologically normal women (Table 3) and discrepancy analysis revealed that 56 true-negative samples were false-positive by Cervista. Therefore, we also calculated HPV positivity as detected by the Cervista HPV HR test using two different modified cut-off criteria (Table 7), which were selected for the detection of CIN3+ lesions. The manufacturer-recommended cut-off is a Fold-Over-Zero (FOZ) ratio of ≥1.525 or <1.525 in combination with an individual-reaction FOZ value of >1.93 if all three probe sets yielded a positive test result. We here examined the cut-off FOZ ratio ≥1.525 without any additional criteria and a FOZ ratio of ≥1.525 or <1.525 with an individual-reaction FOZ value of ≥4.0. As shown in Table 7, HPV prevalence measured by the Cervista HPV HR test in the LBC normal category decreased to 6.7% and 6.1%, respectively, whereas the kappa values increased to 0.73 when using the two different FOZ cut-off values. Relative sensitivity for the detection of CIN3+ was not affected by the modification of the FOZ-values.

**Table 7:** **Different cut-off values for Cervista HPV HR test in LBC-normal samples**

CERVISTA cut-off				
FOZ ratio	FOZ value	CER+ve %	HC2+ve %	κ (95% CI)
≥1.525 or <1.525	≥1.93	10.8%	5.7%	0.52 (0.42-0.61)
≥1.525		6.7%	5.7%	0.73 (0.64-0.81)
≥1.525 or <1.525	≥4	6.1%	5.7%	0.73 (0.65-0.81)

+ve: positive.

## Discussion

This study compares the Cervista HPV HR test to the HC2 test within a German routine cervical cancer screening population of women aged ≥30 years initially screened by LBC. Overall the HR HPV prevalence was 27.8% and 25.2% for Cervista HPV HR and HC2, respectively. Due to the inclusion of 60 additional patients with CIN3+, the overall prevalence is higher than HR HPV prevalence rates of ~9% previously reported for a comparable screening population with participants aged ≥30 years [14]. The overall agreement of the HPV-tests was excellent (κ = 0.8). Relative performance of each HPV test was assessed by calculating relative sensitivity and specificity based on histologically confirmed CIN3+ as “positive” clinical outcome. The relative sensitivities determined for both tests in women with abnormal cytology (AGUS+) were comparable, with 96.6% for Cervista and 98.9% for HC2. Although relative specificity was better for the Cervista HPV HR test (15.7%) than for HC2 (9.8%), the difference was not significant (p value = 0.14), which is supported by the results obtained from our ratio analysis. Our findings confirm the results of previous publications, which compared the performances of the Cervista HPV HR test and the HC2 tests and showed no significant differences in sensitivities detecting histologically confirmed high grade lesions (CIN2+ or CIN3+) [12],[16],[17]. These studies also demonstrated differences in specificities in favour for the Cervista® HPV HR test, however only Belinson et al. was able to show that this difference was of statistical significance [17].

Interestingly, we found a significant difference between HR HPV prevalences detected by Cervista and HC2 in the cytology normal (NILM; negative for intraepithelial lesion or malignancy) group (p < 0.0001; Table 3). In order to resolve discordant results genotyping by INNO-LiPA Extra was performed. LiPA genotyping has previously been used as an adjudicating assay in test comparison studies [26],[27] because of its excellent analytical sensitivity of 20 to 70 copies per assay [28]. In line with previous studies (summarized by [29]) our discrepancy analysis demonstrated substantial cross-reactivity of HC2 with non-target HPV types (n = 16 versus n = 3 for HC2 and Cervista, respectively). Furthermore, discrepancy analysis revealed a high false positive rate for Cervista using the currently approved cut-off values (n = 56 for Cervista versus n = 3 for HC2; Table 5). This result, however, is not in line with previous studies demonstrating a tendency of a lower or no difference in the HR HPV prevalence rate for Cervista in comparison to HC2 in women with NILM cytology [12]-[17]. This disagreement with previous reports might, however reflect differences in study design [12],[13],[16],[17], age range of participants [12],[13],[15]-[17] or overall HR HPV prevalence rates [14]. Nonetheless, our data confirm a commentary, which critically discusses the high false positive detection rate of the Cervista HPV HR test in cytologically normal specimens of women ≥ 30 years of age by evaluating data published with the Cervista HPV HR test package insert [30]. By applying modified cut-off values (increase of the individual-reaction FOZ value from ≥1.93 to ≥4 or the elimination of the individual-reaction FOZ value), which were optimised for the detection of CIN3+ lesions and not HPV DNA in cytologically normal samples, we were able to dramatically reduce the false-positive rate of the Cervista HPV HR test in the Pap normal group resulting in a similar HR HPV-prevalence as detected by HC2, while the relative sensitivity for the detection of high grade disease (CIN3+) remained unaltered. Therefore, a re-adjustment of the manufacturer’s cut-off values for the use of the Cervista HPV HR test in primary screening may be considered, but further population-based studies are necessary to confirm our finding. Studies evaluating the company set cut-off values for the detection of disease have previously been performed for other HPV tests including HC2 where adjustment led to an increased specificity of disease detection and fewer false-positive test results [31]. It is worth noting that current clinical routine in Germany requires ancillary HPV testing only for patients with abnormal or borderline cytology results (PapIIw, PapIII and PapIIID). Importantly, as around 50% of ASC-US specimens will be tested HR HPV positive [32], the accurate early detection of relevant infections by non-invasive and cost-effective tests is fundamental. For the ASC-US category, we demonstrate that the performance of the Cervista HPV HR test was non-inferior to HC2 and rather showed a higher detection rate than HC2. This is in line with previous studies demonstrating a high sensitivity for HR HPV detection and a lower false positive rate as compared to HC2 in ASC-US specimens [13],[14],[18],[19],[21]. Finally, split sample test comparison results of multiple HPV tests other than Cervista have recently been published from the Horizon study where the authors found substantial disagreement between HPV tests especially in women aged 30–65 years. These disagreements were attributed to screening false positives and were probably based on the different assay designs [33].

## Conclusions

We evaluated the performance of the Cervista HPV HR test using residual LBC specimens of a German routine cervical cancer screening population. We demonstrate that the Cervista HPV HR test would be appropriate to be used in primary routine cervical cancer screening after cut-off adjustment or in its current format adjunctive to cytology for detection of high grade cervical intraepithelial neoplasia in women with abnormal (ASC-US+) cytology, yielding similar HR HPV detection rates and relative sensitivity and specificity as the HC2 test. The presence of an internal control as provided by the Cervista HPV HR assay might be responsible for the reduced false negative test results within discrepant samples, as compared to the HC2 test system, which lacks such a control. Another major advantage of the Cervista HPV HR test is the automated integrated DNA extraction step using the HTA system from Hologic, which avoids extra working steps resulting in a minimised risk of sample contamination prior to testing.

## Additional file

## Electronic supplementary material

Additional file 1: Supplemental methods. (PDF 231 KB)

Below are the links to the authors’ original submitted files for images.

Authors’ original file for figure 1

Authors’ original file for figure 2
