# Supplementary material for: A population-based observational study comparing Cervista and Hybrid Capture 2 methods: improved relative specificity of the Cervista assay by increasing its cut-off
Source: BMC Infect Dis. 2014 Dec 9;14:674. doi: 10.1186/s12879-014-0674-1 (PMC4279999; doi:10.1186/s12879-014-0674-1)
Supplement: Supplementary file 1 — Additional file 1: Supplemental methods. (PDF 231 KB) [file 12879_2014_674_MOESM1_ESM.pdf]

A population-based observational study comparing Cervista and Hybrid Capture 2 methods: Improved relative specificity of the Cervista assay by increasing its cut-off

Gerd Boehmer, Lisa Wang, Angelika Iftner, Barbara Holz, Juliane Haedicke, Reinhard von Wasielewski, Peter Martus and Thomas Iftner

SUPPLEMENTAL METHODS

Statistical Analysis of Sensitivity and Specificity

The purpose of the study is the comparison of the sensitivity and specificity criteria between Cervista HPV Assay (CER) and HR HPV HC2 Assay (HC2) in identifying women with abnormal cervical cellular histology. In order to perform a comparison of the tests, one must have a test that verifies the performance of the two tests in question, CER and HC2. In this case, the "gold standard" or verification method used is histology.

However, due to the method of data collection and the ethics of obtaining a histology sample for every patient, there exist histology results for only the patients who have tested positive in the liquid based cytology screening (LBC); a cytology result of greater than or equal to PapIII is considered positive. Furthermore, the likelihood of having a histology result for the patient depends on the Pap screening result, the higher the number for a positive test, the more likely there exists a histology test. Patients with relatively weak but positive LBC results were also unlikely to have a histology result. Thus, it is important to note that there exists very few gold standard data points for patients of negative cytology and varying degrees of incompleteness for patients with positive cytology. In general, patients with $<$ Pap III+ cytology results had a large portion if not all of their Histology results missing. In the following 3 tables, one can see that there was almost no information regarding false negative rates for CER (11 values out a possible of 1123), and 128 histology values out of a total of 484.

TABLE 1. Summary of Histology data for Cervista Study by LBC and CER results

LBC, CER	Hist +	Hist -	Hist NA
LBC+, CER +	85	43	206
LBC+, CER -	3	8	123
LBC-, CER +	0	0	150
LBC-, CER -	0	0	1123

TABLE 2. Summary of Histology data for Cervista Study by LBC and HC2 results

LBC, HC2	Hist +	Hist -	Hist NA
LBC+, HC2 +	87	46	219
LBC+, HC2 -	1	5	110
LBC-, HC2 +	0	0	87
LBC-, HC2 -	0	0	1187

TABLE 3. Proportion of Patients with Histology Results, Stratified by Cytology Results , N=1741

LBC	N	Number with Histology	% with Histology
Pap I/II	1208	0	0%
Pap IIw	65	0	0%
Pap III	20	6	30%
Pap IIID	372	60	16.1%
Pap IVa	71	68	95.8%
Pap IVb	2	2	100%
Pap V	3	3	100%

Methods

When calculating sensitivity or specificity, the data must be divided into the following four non-intersecting categories: True Positive (TP), True Negative (TN), False Positive (FP), False Negative (FN).

Table 4. Data Subdivision

	Gold Std +	Gold Std -
Test +	TP	FP
Test -	FN	TN

Sensitivity.

$$\begin{aligned}\frac{Sens_1}{Sens_2} &= \frac{\frac{TP_1}{TP_1+FN_1}}{\frac{TP_2}{TP_2+FN_2}} \\ \frac{TP+FN}{N_{total}} &= Prevalence \\ Prevalence * N_{total} &= TP+FN\end{aligned}$$

If one seeks to find the sensitivity value for a diagnostic test, then information regarding False Negatives are imperative. However, investigating the ratios of the sensitivity values of two different tests as a means of comparison requires only knowledge of the the True Positive if the tests on the same data set for the same disease. By substituting the equations into the first statement, we have the following:

$$\frac{Sens_1}{Sens_2} = \frac{\frac{TP_1}{Prevalence*N_{total}}}{\frac{TP_2}{Prevalence*N_{total}}} = \frac{TP_1}{TP_2}$$

In this case, True Positives by Cervista number at 85 patients, and 87 patients by HC2. Thus, $\frac{Sens_{car}}{Sens_{hc2}} = 0.977$, with standard error of 0.148. Thus, the 95% CI is (0.69, 1.27).

Specificity.

$$\begin{aligned}
\frac{Spec_1}{Spec_2} &= \frac{\frac{TN_1}{TN_1+FP_1}}{\frac{TN_2}{TN_2+FP_2}} \\
\frac{TN_1+FP_1}{N_{total}} &= 1-Prevalence \\
(1-Prevalence)*N_{total} &= TN+FP
\end{aligned}$$

By substituting the equations into the first statement, we have the following:

$$\frac{Spec_1}{Spec_2} = \frac{\frac{TN_1}{(1-Prevalence)*N_{total}}}{\frac{TN_2}{(1-Prevalence)*N_{total}}} = \frac{TN_1}{TN_2}$$

If we work with the proportions, thus percentage of patients with characteristic, "True Negative" for example, then we have the following equations:

$$\begin{aligned}
TrueNegative &= AllNegative - FalseNegative \\
&= AllNegative - (Prevalence - TruePositive) \\
&= AllNegative - Prevalence + TruePositive \\
&= AllNegative + TruePositive - Prevalence
\end{aligned}$$

Now, using Cervista as an example, note that "All Negative" are all patients that tested negative by the test Cervista, and "True Positive" are all patients that tested positive by both Cervista and Histology.

$$TN_{CER} = \frac{1257 + 85}{1741} - Prevalence = 0.771 - Prevalence$$

$$TN_{HC2} = \frac{1302 + 87}{1741} - Prevalence = 0.798 - Prevalence$$

$$\frac{Spec_{CER}}{Spec_{HC2}} = \frac{0.771 - Prevalence}{0.798 - Prevalence}$$

FIGURE 1. Ratio of Cervista and HC2 Specificity as function of Prevalence, with 95% CI

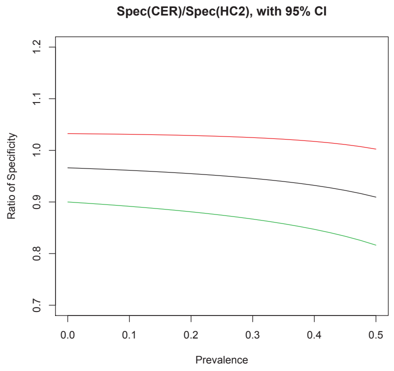

Please see the Appendix for the methods concerning confidence interval calculation.

Conclusion

The calculations did not result in any statistically significant findings due to the amount of variance and unknown within the system. Thus, one cannot say that there exists a difference between Cervista relative to HC2. However, it should be noted here that calculations were made with only the true and false positives known. Especially, in our analysis,

we assumed that the availability of the gold standard is not related to the occurrence of wrong test results.

From the methodological point of view, it is of interest that it was possible to calculate a measure of relative sensitivity and specificity even while the performance of the test for negative results is unverified. This was possible because both tests were evaluated in the same subjects, and thus the unknown prevalences were exactly equal for both tests.

Appendix

Confidence Intervals. It is reasonable to view the count data of TP or other categories are events from a Poisson distribution. Due to the large sample size, we can also assume normal approximation. Thus, if $X \sim Poisson(\lambda)$, then under large sample sizes with a small event rate, we can say $X \sim Normal(\lambda, \lambda)$. Thus, applying the δ-method on the ratios, we can calculate the variance of the ratios:

$$\begin{aligned} g(x,y) &= \frac{x}{y} \\ Var(\frac{x}{y}) &= \frac{1}{\mu_y^2}Var(x) + \frac{\mu_x^2}{\mu_y^4}Var(y) - 2\frac{\mu_x}{\mu_y^3}Cov(x,y) \end{aligned}$$

Using the above equations, we apply the δ-method to the sensitivity and specificity ratios, with the equations simplifying to:

$$\begin{aligned} \hat{Var}(\frac{Sens_{CER}}{Sens_{HC2}}) &= \frac{\hat{Var}(TP_{CER})}{\hat{Var}(TP_{HC2})^2} + \frac{\hat{Var}(TP_{CER})^2}{\hat{Var}(TP_{HC2})^3} \\ &- 2\frac{\hat{Var}(TP_{CER})}{\hat{Var}(TP_{HC2})^3}\hat{Cov}(TP_{CER}, TP_{HC2}) \end{aligned}$$

Similarly, we apply the δ-method to the specificity ratios. When doing so, we can convert the numbers to count data by renormalizing with the population size:

$$\begin{aligned}\frac{Spec_{CER}}{Spec_{HC2}} &= \frac{0.771 - Prevalence}{0.798 - Prevalence} \\ &= \frac{1257 + 85 - (Prevalence * 1741)}{1302 + 87 - (Prevalence * 1741)}\end{aligned}$$

Now, taking the above ratio, we can use the variance formula of $Var(\frac{Sens_{CER}}{Sens_{HC2}})$, except now we can write $Var(\frac{Spec_{CER}}{Spec_{HC2}})$. By assuming prevalence is some unknown but fixed number between 0 and 1, we can calculate the confidence interval point wise for a range of prevalence values.
