# Supplementary figures and images for: A population-based observational study comparing Cervista and Hybrid Capture 2 methods: improved relative specificity of the Cervista assay by increasing its cut-off
Source: BMC Infect Dis. 2014 Dec 9;14:674. doi: 10.1186/s12879-014-0674-1 (PMC4279999; doi:10.1186/s12879-014-0674-1)

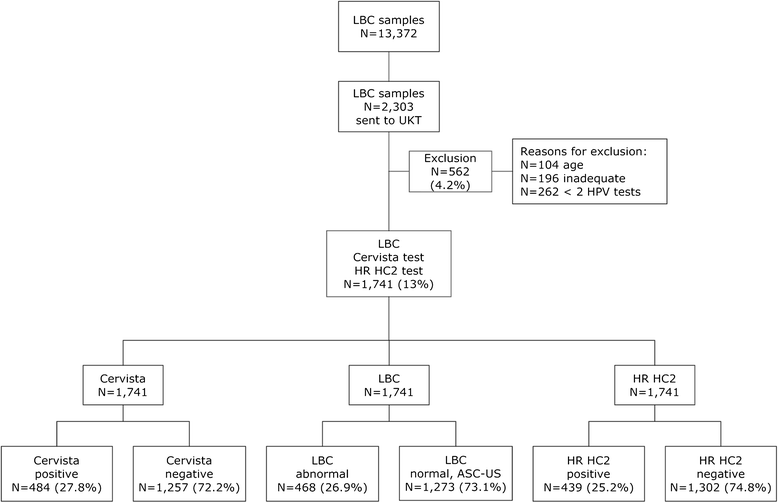

Supplement: Supplementary file 2 — Authors’ original file for figure 1 [file 12879_2014_674_MOESM2_ESM.gif]

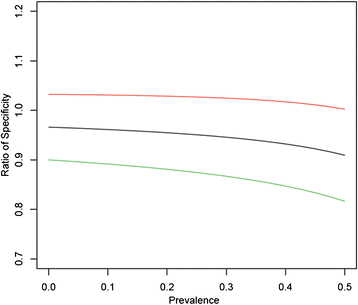

Supplement: Supplementary file 3 — Authors’ original file for figure 2 [file 12879_2014_674_MOESM3_ESM.gif]
